# Pharmacological Modulation of Human Mesenchymal Stem Cell Chondrogenesis by a Chemically Oversulfated Polysaccharide of Marine Origin: Potential Application to Cartilage Regenerative Medicine

**DOI:** 10.1002/stem.1686

**Published:** 2011-11-30

**Authors:** Christophe Merceron, Sophie Portron, Caroline Vignes-Colombeix, Emilie Rederstorff, Martial Masson, Julie Lesoeur, Sophie Sourice, Corinne Sinquin, Sylvia Colliec-Jouault, Pierre Weiss, Claire Vinatier, Jérôme Guicheux

**Affiliations:** a Institut National de la Santé et de la Recherche Médicale (INSERM), UMRS 791 Laboratoire d'Ingénierie Ostéo-Articulaire et Dentaire, Group STEP “Skeletal Tissue Engineering and Physiopathology,” Université de NantesNantes, France; b PRES-UNAM, UFR Odontologie, Université de NantesNantes, France; c IFREMER, Laboratoire de biotechnologie et molécules marines (BRM/BMM)Nantes, France; d GRAFTYS SA, Pôle d'Activités d'Aix en ProvenceAix en Provence, France

**Keywords:** Cartilage, Adipose-derived mesenchymal stem cells, Polysaccharides, Transforming growth factor-β

## Abstract

Mesenchymal stem cells (MSCs) are considered as an attractive source of cells for cartilage engineering due to their availability and capacity for expansion and multipotency. Differentiation of MSC into chondrocytes is crucial to successful cartilage regeneration and can be induced by various biological agents, including polysaccharides that participate in many biological processes through interactions with growth factors. Here, we hypothesize that growth factor-induced differentiation of MSC can be increased by chemically oversulfated marine polysaccharides. To test our hypothesis, human adipose tissue-derived MSCs (hATSCs) were cultured in pellets with transforming growth factor (TGF)-β1-supplemented chondrogenic medium containing either the polysaccharide GY785 DR or its oversulfated isoform GY785 DRS. Chondrogenesis was monitored by the measurement of pellet volume, quantification of DNA, collagens, glycosaminoglycans (GAGs), and immunohistological staining. Our data revealed an increase in pellet volume, total collagens, and GAG production with GY785 DRS and chondrogenic medium. The enhanced chondrogenic differentiation of hATSC was further demonstrated by the increased expression of several chondrogenic markers by real-time reverse transcription-polymerase chain reaction. In addition, surface plasmon resonance analyses revealed that TGF-β1 bound GY785 DRS with higher affinity compared to GY785 DR. In association with TGF-β1, GY785 DRS was found to upregulate the phosphorylation of extracellular signal-regulated kinase 1/2, indicating that oversulfated polysaccharide affects the mitogen activated protein kinase signaling activity. These results demonstrate the upregulation of TGF-β1-dependent stem cell chondrogenesis by a chemically oversulfated marine polysaccharide. This polysaccharide of marine origin is easily producible and therefore could be considered a promising additive to drive efficient and reliable MSC chondrogenesis for cartilage tissue engineering. Stem Cells *2012;30:471–480*

## INTRODUCTION

Articular cartilage is a specialized tissue that surrounds the ends of long bones. It reduces friction and acts as shock-absorbing tissue during joint mobilization. Cartilage is composed of a single cell type, the chondrocyte, that is responsible for the synthesis of an abundant extracellular matrix (ECM) essentially composed of type II, IX, and XI collagens and proteoglycans [1, 2]. Chondrocytes play a major role in maintaining the integrity of cartilage through the control of anabolic and catabolic processes. However, cartilage is susceptible to damage through aging, trauma, and inflammatory or degenerative diseases. These impairments often result in ECM degradation and ultimately in the loss of joint function.

Many surgical approaches have been developed to improve the poor intrinsic self-repair capacity of cartilage. Unfortunately, these techniques have not shown satisfactory efficacy. In this context, the regeneration of functional cartilage through the transplantation of mesenchymal stem cells (MSCs) with bioactive synthetic matrices has recently been contemplated [3, 4]. MSCs were initially identified in bone marrow [5] and since have been detected in various other tissues such as adipose tissue, tendon, synovial membrane, muscle, and periosteum [6]. MSCs exhibit a number of attractive features, particularly for regenerative medicine, including their self-renewal, their ability to proliferate in culture, and their multipotency [7]. Biological sulfated polysaccharides such as glycosaminoglycans (GAGs) have been shown to modulate the biological activities of growth factors including transforming growth factor (TGF)-β [8, 9]. The binding of growth factors to GAG can increase their local concentration up to levels appropriate for signaling, protect them from degradation by extracellular proteases, and act as coreceptors, facilitating their interaction with their receptors [10]. The actions of biologically active polysaccharides are largely dependent on their molecular structure, in particular their molecular size and varying size of repeating unit features, osidic residues, linkage, and sulfation degree.

Natural polysaccharides derived from marine prokaryotes offer a significant structural chimiodiversity with novel, striking biological properties [11, 12]. In addition, natural GAG-mimetics can be chemically customized and produced in large amounts with relatively simple and reproducible processes, making them potentially suitable as bioactive agents for medical applications. Among the large number of prokaryotic species capable of producing GAG [11], *Alteromonas infernus* has been shown to produce a branched high-molecular weight polysaccharide: GY785 (∼10^6^ g/mol). This polysaccharide is unique with no known analog [13]. Low-molecular weight (GY785 DR) and low-molecular weight oversulfated (GY785 DRS) derivatives of GY785 have been produced by chemical modification and described as “heparin-like” compounds, exhibiting some anticoagulant and proangiogenic properties [13, 14].

With respect to the potential effects of GAG-like polysaccharides on the regulation of growth factor bioavailability, we postulated that low-molecular weight marine polysaccharides such as oversulfated or standard GY785 may influence the growth factor-mediated chondrogenic differentiation of MSC.

To address this issue, we sought to determine whether the presence of oversulfated or standard GY785 could influence the chondrogenic differentiation of human adipose tissue-derived MSC (hATSC) driven by a TGF-β1 and insulin-supplemented culture medium. We first assessed the effects of GY785 DR and GY785 DRS on hATSC viability and proliferation. Thereafter, we investigated the effect of these two polysaccharides on the chondrogenic differentiation of hATSC pellets by determining the volume and the DNA, total collagens, and GAG contents before immunohistological characterization of the pellets. At a transcriptional level, mRNA encoding the *COL2A1*, *ACAN*, *COMP*, *SOX9*, *COL1A1*, and *COL10A1* were analyzed by real-time reverse transcription-polymerase chain reaction (RT-PCR). Physical interactions between chondrogenic (CH) medium constituents (TGF-β1 and insulin) and polysaccharides were investigated using the surface plasmon resonance approach. To obtain further insight into the regulation of cell signaling, we additionally studied the effect of GY785 DRS on the major signaling pathways activated by TGF-β1 in hATSC.

## MATERIALS AND METHODS

### Materials

Cell culture plastic wares were purchased from Corning-Costar BV Life Sciences (Schipol-Rijk, The Netherlands, http://www.corning.com/lifesciences/emea/en/index.aspx). Hank's Balanced Sodium Salt (HBSS), Dulbecco's modified Eagle's medium (DMEM) high glucose (4.5 g/L), alpha minimum essential medium, phosphate-buffered salt (PBS), penicillin/streptomycin, trypsin/EDTA (0.05%/0.53 mM), L-glutamine, superscript III kit, NuPAGE 4%–12% Bis–Tris gel, bone morphogenetic protein-2 (BMP-2), and polyvinylidene difluoride (PVDF) Invitrolon membranes were obtained from Invitrogen Corporation (Paisley, UK, http://www.invitrogen.com). Vascular endothelial growth factor (VEGF) was purchased from Amrad Corporation (Richmond, Australia, http://www.amrad.com.au). Low-molecular weight heparin (4,500 g/mol) was obtained from Sanofi-Aventis (Paris, France, http://www.sanofi.com/). 3-(4,5-Dimethylthiazol-2-yl)-5-(3-carboxymethoxyphenyl)-2-(4-sulfophenyl)-2H-tetrazolium, inner salt (MTS) reagents were from Promega (Charbonnières, France, http://www.promega.com/products). Collagenase crude type I A; red blood cell lysis buffer; trypan blue; sodium L-ascorbate; insulin, transferrin, selenite media supplement; dexamethasone; Alcian Blue; papain; pepsin; anisomycin; and DNA quantification kit were purchased from Sigma-Aldrich (St. Louis, MO, http://www.sigmaaldrich.com/france.html). Total collagens (Sircol) and GAG (Blyscan) quantification kits were from Biocolor (Carrickfergus, UK, http://www.biocolor.co.uk). Brilliant SYBR Green Master Mix was obtained from Stratagene Europe (Amsterdam Zuidoost, The Netherlands, http://www.stratagene.com). PCR primers were synthesized by MWG Biotech (Ebersberg, Germany, http://www.mwg-biotech.com). Fetal calf serum (FCS) was purchased from Dominique Dutscher (Brumath, France, http://www.dutscher.com). TGF-β1 and Insulin-like growth factor-1 (IGF-1) were obtained from PeproTech Inc. (London, UK, http://www.peprotechec.com). The RNeasy micro kit was purchased from Qiagen (http://www.qiagen.com) and turbo DNase from Ambion Inc. (http://www.invitrogen.com/site/us/en/home/brands/ambion.html), both distributed by Applied Biosystems (Courtaboeuf, France, http://www.appliedbiosystems.com). Protein content was determined using the Pierce Coomassie Plus assay (Pierce, Rockford, IL, http://www.piercenet.com). The rabbit anti-phospho-SMAD 2 (3101), phospho-extracellular signal-regulated kinase (ERK)1/2 (9101), phospho-c-Jun N-terminal kinase (JNK)1/2 (9251), phospho-p38 (9211), SMAD 2 (3102), ERK1/2 (9102), and goat anti-rabbit IgG horseradish peroxidase (HRP)–linked (7074) antibodies were purchased from Cell Signaling Inc. (Beverly, MA, http://www.cellsignal.com/index.jsp). The Western blot detection system was obtained from GE Healthcare (Buckin-ghamshire, UK, http://www3.gehealthcare.com). Anti-human type I (631701) and type II (08631711) collagens monoclonal mouse antibodies were purchased from MP Biomedicals Europe (Illkirch, France, http://www.mpbio.com). Monoclonal antibody directed against human type X collagen (2031501018) was purchased from Quartett (Berlin, Germany, http://www.quartett.com/products.htm). All other chemicals were obtained from standard laboratory suppliers and were of the highest purity available.

### Production, Purification, and Characterization of GY785 DR and GY785 DRS Polysaccharides

The two low-molecular weight polysaccharides GY785 DR and GY785 DRS were derived from GY785, a high-molecular weight exopolysaccharide (∼10^6^ g/mol) produced by a bacterium, *A. infernus*. The isolation procedure and characterization of the strain *A. infernus* have previously been described by Raguenes et al. [15]. The native polysaccharide can undergo a radical depolymerization to obtain GY785 DR. Then, the GY785 DR can be chemically oversulfated to obtain the GY785 DRS as described elsewhere [16]. GY785 DR and GY785 DRS are homogenous fractions with average molecular masses of 15 × 10^3^ g/mol and 20 × 10^3^ g/mol and 10% and 40% sulfate groups, respectively, as determined by analytical high performance size-exclusion chromatography (Supporting Information Fig. 1) [13].

### Cell Culture

hATSCs were isolated by collagenase digestion of lipoaspirates obtained from three different patients undergoing liposuction and who had given their informed consent. All protocols were approved by the French national ethical committee. Briefly and as previously described [17, 18], lipoaspirates were washed extensively with HBSS to remove debris and then treated with collagenase (0.025%) in HBSS for 1 hour at 37°C under gentle agitation. The collagenase treatment was inactivated by adding an equal volume of DMEM high glucose containing 1% penicillin/streptomycin, 1% L-glutamine, and 10% FCS (control [CT] medium). The digested product was then centrifuged at 250*g* for 5 minutes to separate adipocytes from stromal cells. The supernatant was removed and cells were resuspended in the control medium and filtered through a 70-μm nylon mesh filter. The filtrate was centrifuged and cells were resuspended in red blood cell lysis buffer. The lysis reaction was stopped by adding control medium. The suspension was centrifuged and cells were finally resuspended in control medium and plated at 5 × 10^4^ cells per cm^2^ in culture flasks. Cells were incubated at 37°C in a humidified atmosphere and the control medium was replaced 24 hours after seeding to remove nonadherent cells. Thereafter, the control medium was renewed every 2-3 days. hATSC primary cultures were grown to 90% confluence and then detached from the culture flask using trypsin/EDTA, then replated at 1 × 10^4^ cells per cm^2^. For all subsequent experiments, hATSCs were used at passage 2. hATSCs isolated using the above-described protocol have been extensively characterized in our laboratory (for details [18, 19]).

### Viability and Proliferation

Cell viability was evaluated using an MTS assay as previously described [20]. hATSCs were plated at a density of 1 × 10^4^ cells per cm^2^ and cultured in control medium in the absence or presence of 25, 50, and 100 μg/ml of GY785 DR or GY785 DRS for 72 hours. As a negative control, cells were cultured in the presence of actinomycin D (5 μg/ml) a well-known cell death inducer [20]. Results were expressed as relative MTS activity compared to the control condition (cells cultured in the absence of polysaccharide).

To correlate MTS activity with cell proliferation, we also estimated the number of viable cells. As previously described, cells were treated with trypsin/EDTA after 72 hours of culture and counted using trypan blue exclusion dye [18, 20]. Results were expressed as the total number of viable cells per square centimeter compared with the control condition (cells cultured in the absence of polysaccharide).

### Chondrogenic Differentiation

For the in vitro chondrogenic differentiation of hATSC, 5 × 10^5^ cells were placed into polypropylene tubes containing 1 ml of control medium, as previously reported [18]. They were then centrifuged for 5 minutes at 250*g*. The tubes were fitted with vented caps to permit gas exchange, and the cell pellets were maintained at 37°C in a humidified atmosphere. Pellets of hATSCs were divided into seven experimental groups and cultured for the indicated times either in the presence of CT or CH medium alone or supplemented with 50 μg/ml or the indicated concentrations of GY785 DR, GY785 DRS, or heparin. The chondrogenic medium was composed of serum-free control medium supplemented with 6.25 μg/ml insulin, 6.25 μg/ml transferin, 6.25 ng/ml sodium selenite; 50 nM sodium L-ascorbate; 10^−8^ M dexamethasone; and 10 ng/ml TGF-β1 [18, 19]. Culture media were changed every 2-3 days.

### Human ATSC Pellet Characterization

For the measurement of pellet volume, pellets were considered as scalene ellipsoid entities. Their volumes were estimated using the following formula:


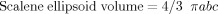


In which *a*, *b*, and *c* represent the radius of long axis in each spatial plane.

For pellet gross appearance, the production of sulfated GAG was investigated on whole 28-day-old pellets by Alcian Blue staining. Pellets were washed with ice-cold PBS and fixed for 20 minutes in 100% ethanol. Pellets were then stained with 0.1% Alcian Blue solution in 0.1 M HCl. After overnight incubation, the solution was discarded and the pellets were rinsed with 0.1 M HCl to eliminate nonspecific staining. Photographs were obtained with a stereomicroscope (MZ6, Leica, Wetzlar, Germany, http://www.leica-microsystems.com).

For DNA and GAG quantification, pellets were digested by papain solution (2 mg/ml) for 3 hours at 60°C. DNA content was determined using the DNA binding fluorochrome bisbenzimide following manufacturer's instructions. DNA amount was evaluated using a standard curve of calf thymus DNA. GAG production was determined using a Blyscan Kit according to the manufacturer's indications. For total collagens quantification, pellets were minced and digested overnight by pepsin solution (0.1 mg/ml) at 4°C. Isolation and quantification of collagen were performed using Sircol Kit according to the manufacturer's instructions.

For histological and immunohistological analysis, hATSC pellets were fixed in 10% formalin and embedded in paraffin. Paraffin sections (5-μm thick) were deparaffinized using toluene, rehydrated through a graded series of ethanol, and rinsed in distilled water. Tissue sections were stained with hematoxylin-eosin-safran (HES) and Alcian Blue and immunostained for type I, II, and X collagens as previously described [18]. As negative control, sections were processed using identical protocols, but omitting the primary antibody. Sections were then visualized using a light microscope (Axioplan 2, Zeiss, Göttingen, Germany, http://www.zeiss.com). Alcian Blue reveals the presence of a GAG-containing cartilaginous matrix, HES stains the nucleus in purple, the cytoplasm in pink, and collagen fibers in yellow and immunopositive areas exhibit brown staining.

### Transcript Analysis

Total RNA from hATSC pellets was extracted using the RNeasy micro kit in accordance with the manufacturer's instructions. After DNase digestion, RNA was quantified using a UV-spectrophotometer (Nanodrop ND-1000, Labtech, Palaiseau, France, http://www.nanodrop.com) and quality was determined with the Agilent Bioanalyser 2100 system (Waldbronn, Germany, http://www.genomics.agilent.com). Five hundred nanograms of RNA per sample were reverse transcribed using the superscript III kit in a total volume of 30 μL. Complementary DNA (cDNA) was amplified in a total volume of 25 μL PCR reaction mix containing 12.5 μL of Brilliant SYBR Green Master Mix (1×) and 30 nM SYBR green reference dye. The sequences of each primer set are provided in Supporting Information Table 1. The real-time polymerase chain reaction was carried out in a MX3000P real-time PCR system (Stratagene) under the following conditions: 10 minutes at 95°C followed by 40 cycles of 30 seconds at 95°C, 1 minute at 60°C and 30 seconds at 72°C. The efficiency and specificity of each primer set were confirmed with standard curves of cycle threshold (Ct) values versus serial dilution of total RNA and melting profile evaluation. Cycle thresholds were normalized to β-actin to control for cDNA quantification differences. Results were reported as relative expression levels.

### Surface Plasmon Resonance

Experiments were carried out on a Biacore 3000 instrument (Biacore, Uppsala, Sweden, http://www.biacore.com/lifesciences/index.html). TGF-β1, insulin, BMP-2, VEGF, and IGF-1 were covalently immobilized to the dextran matrix of a CM5 sensor chip (Biacore) as recommended by the manufacturer at a flow rate of 5 μL/minute. Binding assays of GY785 DR, GY785 DRS, and heparin were performed in 10 mM HEPES buffer, pH 7.4, containing 0.15 M NaCl and 0.005% P_2_O surfactant [4-(2-hydroxyethyl)-1-piperazineethanesulfonic acid (HEPES)–buffered saline-NaCl: 0.15 M NaCl, 0.01 M HEPES, pH 7.4 with 0.005% vol/vol surfactant P20 (Biacore)] and dissociation was monitored for 15 minutes. Regeneration was achieved with NaOH (4.5 mM) after each cycle. The resulting sensorgrams were fitted using BiaEval 4.1 software (Biacore). For *K*_d_ calculations, the following molecular weights were used: GY785 DR: 15 × 10^3^ g/mol and GY785 DRS: 20 × 10^3^ g/mol.

### Western Blotting

Confluent hATSCs were cultured in the presence of TGF-β1 (10 ng/ml) alone or in combination with GY785 DR or GY785 DRS (50 μg/ml) in control medium containing low serum levels (0.5%) for 1-, 4-, 8-, and 24-hour periods. For each time point, cells were rapidly frozen in liquid nitrogen and conserved at −80°C until use. For Western blotting, cells were thawed on ice and lysed by the addition of RIPA buffer (20 mM Tris-HCl, pH 7.5, 100 mM potassium chloride, 1 mM EDTA, 1 mM EGTA, 1 mM dithiothreitol, 20 mM β-glycerophosphate, 2 mM Na_3_VO_4_, 1 mM phenylmethylsulfonyl fluoride, and 1 mM NaF). The protein concentration of cell lysates was determined with a Pierce Coomassie-Plus-protein assay. Thirty micrograms of total protein were resolved by sodium dodecyl sulfate-polyacrylamide gels, and proteins were transferred to a PVDF membrane following the manufacturer's protocol. Membranes were blocked and probed in 5% nonfat dry milk in PBS/Tween 20. Primary antibodies were diluted 1/1,000 and were detected using goat anti-rabbit (HRP)-conjugated secondary antibodies diluted 1/2,000 in 5% nonfat dry milk in PBS/Tween 20. The blots were visualized by enhanced chemiluminescence development using a Western blotting detection system.

As a positive control for SMAD and mitogen activated protein (MAP) kinase activation, confluent osteoblastic MC3T3-E1 cells were serum starved overnight and treated for 15 minutes with anisomycin (5 μg/ml), TGF-β1 (10 ng/ml), or inorganic phosphate (Pi; 10 mM) as previously described [21].

### Statistical Analysis

Each experiment was repeated at least three times with similar results. Results are expressed as mean ± SEM of triplicate determinations. Comparative studies of means were performed by using one-way analysis of variance followed by a post hoc test (Fisher's projected least significant difference) with a statistical significance at *p* < .05.

## RESULTS

### Viability and Proliferation of hATSC Cultured in the Presence of Polysaccharides

**Figure 1:**
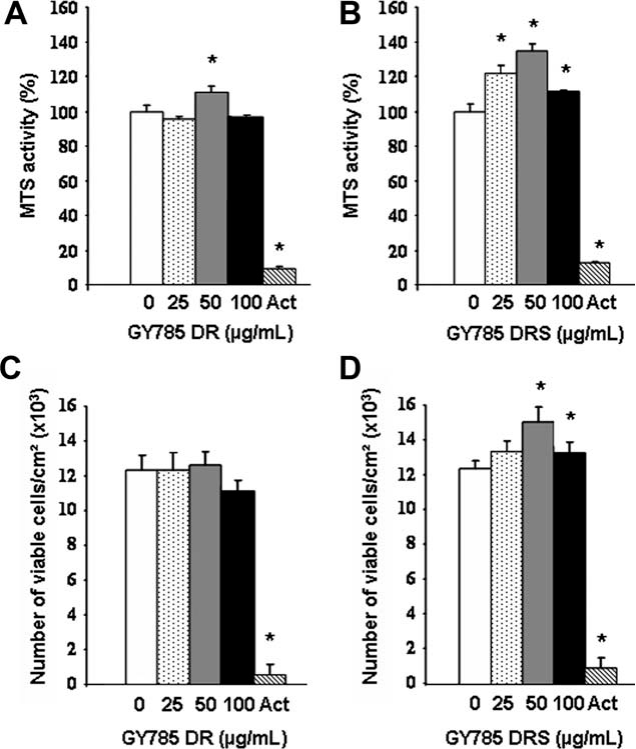
Human adipose tissue-derived mesenchymal stem cell (ATSC) viability and proliferation. Human ATSCs were cultured in the presence of either GY785 DR **(A and C)** and GY785 DRS **(B and D)** at the indicated concentrations or Act (5 μg/ml) for 72 hours. Viability (A and B) was evaluated by MTS activity measurement and expressed as the relative MTS activity compared to the untreated control. Proliferation (C and D) was estimated by viable cell counting after trypan blue exclusion dye. *p < .05 compared to the control condition (0 μg/ml of polysaccharide). Abbreviations: Act, actinomycin D; MTS, 3-(4,5-dimethylthiazol-2-yl)-5-(3-carboxymethoxyphenyl)-2-(4-sulfophenyl)-2H-tetrazolium, inner salt.

To examine the viability and the proliferation of hATSC cultured in the presence of 0, 25, 50, and 100 μg/ml of GY785 DR or GY785 DRS, MTS activity was measured and cells were enumerated using trypan blue exclusion dye after 72 hours. When treated with actinomycin D, the MTS activity and the proliferation of hATSC were reduced by nearly 90%. At the concentration of 50 μg/ml, GY785 DR induced a slight but significant increase in MTS activity (Fig. 1A). GY785 DRS was found to trigger a dose-dependent increase in MTS activity, with a maximum at 50 μg/ml (Fig. 1B). Regarding hATSC proliferation, GY785 DR did not elicit any beneficial effect (Fig. 1C), whereas the sulfated form of the molecule (GY785 DRS) at 50 μg/ml significantly increased hATSC proliferation in comparison to the control condition (Fig. 1D).

### Chondrogenic Differentiation of hATSC Cultured in the Presence of Polysaccharides

**Figure 2:**
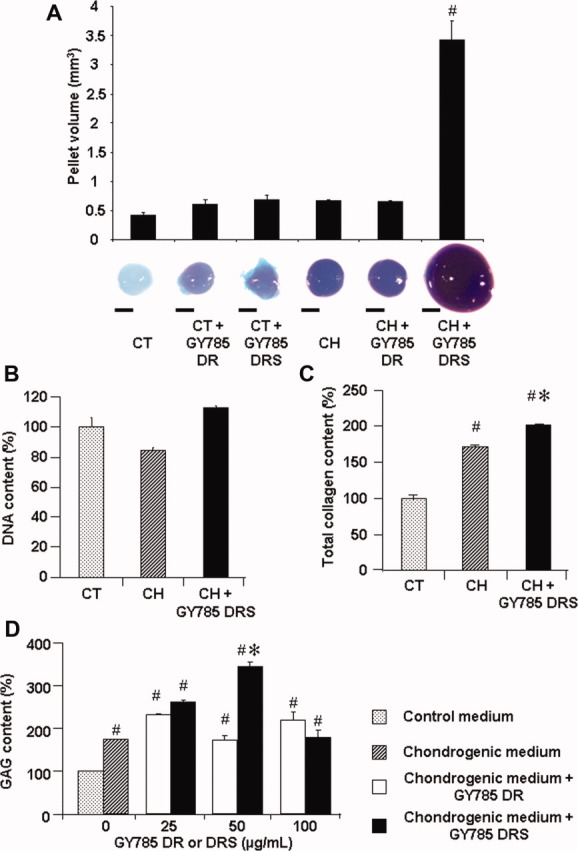
Biochemical characterization of human adipose tissue-derived mesenchymal stem cell (hATSC) pellets. hATSCs were cultured in pellets in the presence of CT or CH medium supplemented or not with 50 μg/ml or the indicated concentrations of GY785 DR or GY785 DRS for 28 days. **(A):** The pellet volumes were calculated by equating these entities to ellipsoids. ^#^*p* < .05 compared to the control condition. GAG production was evaluated at a gross level after Alcian Blue staining of the whole pellets. Bar: 500 μm. **(B):** Total DNA contents were evaluated using a DNA quantification kit. Results are expressed as the relative content compared to the control condition. ^#^*p* < .05 compared to the control condition. *p < .05 compared to the chondrogenic condition. **(C):** Total collagens contents were quantified using a Sircol Kit. Results are expressed as the relative content compared to the control condition. ^#^*p* < .05 compared to the control condition. *p < .05 compared to the chondrogenic condition. **(D):** GAG contents were quantified using a Blyscan Kit. Results are expressed as the relative content compared to the control condition. ^#^*p* < .05 compared to the control condition. *p < .05 compared to the chondrogenic medium supplemented with GY785 DR at the same concentration. Abbreviations: CH, chondrogenic; CT, control; GAG, glycosaminoglycan.

Since GY785 DR and GY785 DRS are considered as GAG-like polysaccharides [13, 14], we paid attention to their putative properties in driving MSC chondrogenic differentiation. To this end, hATSCs were cultured in three-dimensional pellets in the presence of CT or CH medium supplemented with of GY785 DR, GY785 DRS, or heparin for the indicated times. Chondrogenic differentiation was evaluated by the measurement of pellet volume and by the production of a GAG-containing cartilaginous matrix using Alcian Blue staining (Fig. 2A).

Surprisingly, volume estimation revealed that pellets exposed to GY785 DRS in combination with the chondrogenic medium underwent a massive increase by nearly eightfold (Fig. 2A). In addition, to highlight differences in terms of GAG production between the different conditions, the gross appearance of pellets was observed after Alcian Blue staining. Alcian Blue staining revealed that in the presence of chondrogenic medium, and regardless of polysaccharide supplementation, GAG production was upregulated as shown by the intense dark blue color (Fig. 2A). Heparin failed to exert dramatic effect on pellet volume (Supporting Information Fig. 2).

To decipher whether the size increase observed for hATSC pellets cultured in the presence of chondrogenic medium supplemented with GY785 DRS was due to an enhanced cell proliferation or an increased ECM production, DNA (Fig. 2B), total collagens (Fig. 2C), and GAG (Fig. 2D) have been quantified. DNA content was not significantly affected, whereas these data point to a slight but significant increase in the production of total collagens and a twofold increase in the production of GAG. In addition, the dose-response experiment evidenced that the dose of 50 μg/ml of GY785 DRS was the optimal concentration for the increased production of GAG.

**Figure 3:**
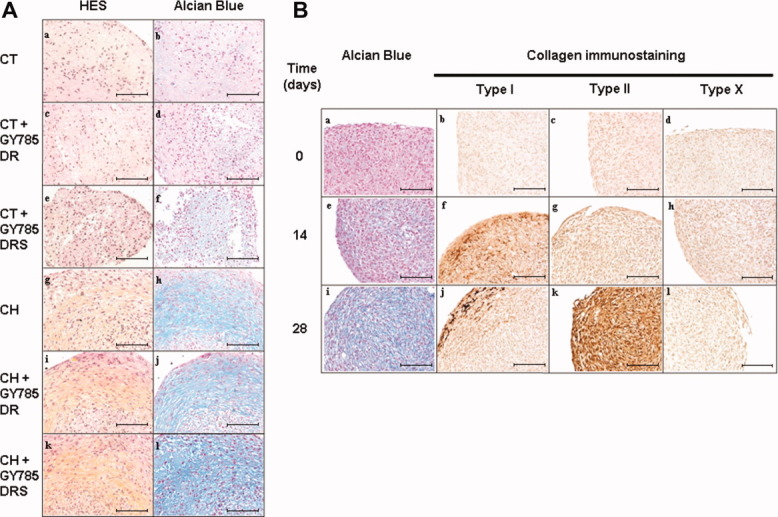
Histological characterization of human adipose tissue-derived mesenchymal stem cell (hATSC) pellets. **(A):** Human ATSCs were cultured in pellets for 28 days in the presence of CT or CH medium supplemented or not with GY785 DR or GY785 DRS (50 μg/ml). Histological sections of hATSC pellets were stained with HES (a, c, e, g, i, and k) and Alcian Blue (b, d, f, h, j, and l) as indicated in Materials and Methods. Bar: 100 μm. **(B):** Human ATSCs were cultured in pellets for 0, 14, and 28 days in the presence of chondrogenic medium supplemented with GY785 DRS (50 μg/ml). Histological sections of hATSC pellets were stained with Alcian Blue (a, e, and i) and immunostained for type I (b, f, and j), II (c, g and k), and X (d, h, and l) collagens as indicated in Materials and Methods. Bar: 100 μm. Abbreviations: CH, chondrogenic; CT, control; HES, hematoxylin-eosin-safran.

In the presence of CT medium, HES staining distinguished cell nuclei (purple) and cytoplasm (pink) but did not reveal any particular organization of the cells (Fig. 3A a, c, and e). On the contrary, in the presence of CH medium, supplemented or not with polysaccharides, HES staining revealed a particular structural organization of the cells within the pellet (Fig. 3A g, i, and k). In the external zone, the cells were tangentially oriented to the surface of the pellet and in the innermost part, the cells seemed to be arranged radially. Moreover, in the presence of chondrogenic medium, a yellow-orange ring is visible, demonstrating the synthesis of collagen fibers within the matrix (Fig. 3A g, i, and k). Alcian Blue staining failed to reveal the presence of sulfated GAG within the matrix of pellets cultured in control medium (Fig. 3A b, d, and f). Interestingly, in the presence of chondrogenic medium, pellet sections were strongly positive for sulfated GAG (Fig. 3A h, j, and l), especially in the presence of chondrogenic medium supplemented with GY785 DRS polysaccharide. For accurate analysis of the nature and kinetics of matrix components synthesis, we performed a time course experiment. A gradual accumulation of GAG within the matrix was demonstrated by Alcian Blue staining (Fig. 3B a, e, and i), suggesting that GY785 DRS affects the chondrogenesis of hATSC in a time-dependent manner. Type I collagen was detected in the outer part of pellet sections from day 14 (Fig. 3B b, f, and j). The production of type II collagen was barely detectable at day 14. By contrast, pellet sections at day 28 exhibited strong positive areas (Fig. 3B c, g, and k). The synthesis of type X collagen could not be detected (Fig. 3B d, h, and l), and this for all the culture conditions tested.

**Figure 4:**
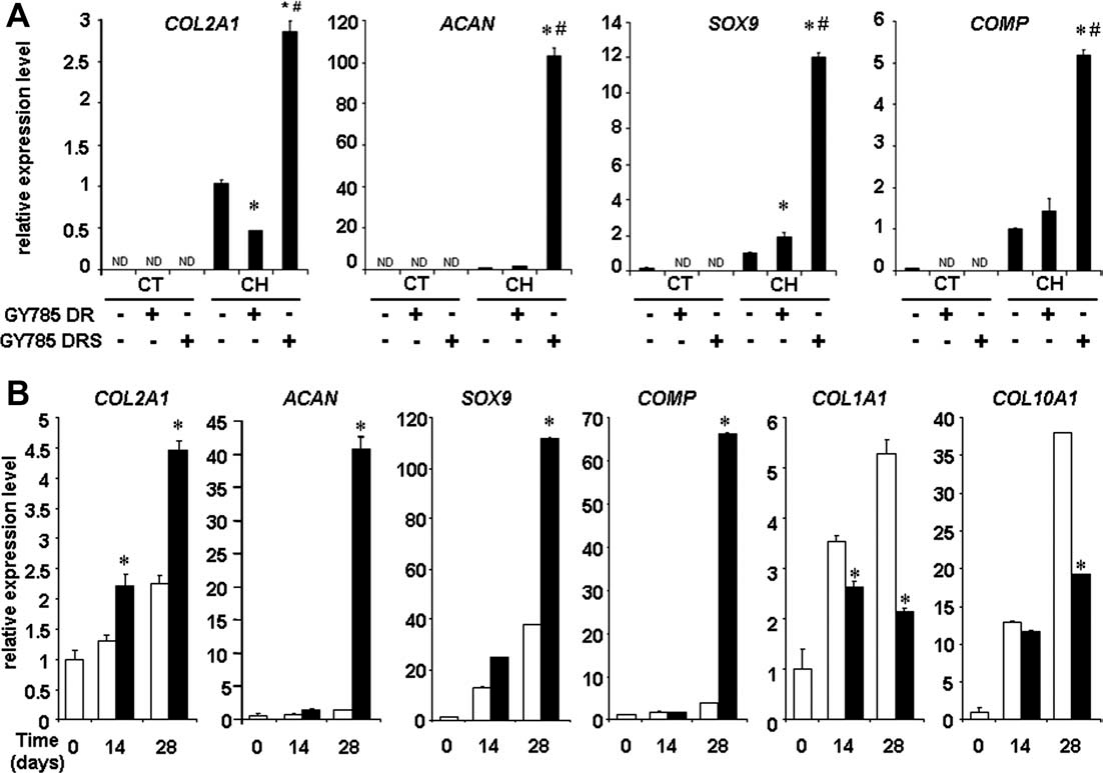
Analysis of the expression levels of chondrogenic markers. **(A):** Human adipose tissue-derived mesenchymal stem cell (ATSC) pellets were cultured in the presence of CT or CH medium supplemented or not with 50 μg/ml of GY785 DR or GY785 DRS for 28 days. Expression of the chondrogenic markers *COL2A1, ACAN, SOX9*, and *COMP* was investigated by real-time reverse transcription-polymerase chain reaction (RT-PCR). Results are expressed as relative expression level compared to the chondrogenic medium in the absence of polysaccharide. *p < .05 compared to chondrogenic medium. ^#^*p* < .05 compared to chondrogenic medium supplemented with GY785 DR. **(B)** Human ATSC pellets were cultured in the presence of chondrogenic medium (white bars) or chondrogenic medium supplemented with 50 μg/ml of GY785 DRS (black bars) for 0, 14, and 28 days. Expression of *COL2A1, ACAN, SOX9, COMP, COL1A1*, and *COL10A1* was investigated by real-time RT-PCR. Results are expressed as relative expression level compared to the basal level at day 0 in the absence of polysaccharide. *p < .05 compared to the chondrogenic condition at the same day. Abbreviations: CH, chondrogenic; CT, control.

To confirm the cartilage-like appearance of hATSC pellets shown by our histological observations, we sought to determine the relative expression of *COL2A1*, *ACAN*, *SOX9*, and *COMP* transcripts, by real-time RT-PCR. Our data indicate that in the presence of CT medium supplemented or not with polysaccharide, transcripts coding for the various chondrocyte markers could not be detected (ND) or remained at barely detectable levels. For each transcript analyzed, the expression level was significantly increased in the presence of chondrogenic medium compared to the control medium. Of interest, GY785 DRS used in combination with chondrogenic medium induced a marked increase in the expression levels of the four chondrogenic markers when compared to GY785 DR (Fig. 4A). Interestingly, in a time course experiment (Fig. 4B), our data confirmed that the effects of GY785 DRS were time-dependent. In addition to promote the expression of the above-mentioned chondrogenic markers, the association of GY785 DRS with the chondrogenic medium significantly reduces the expression levels of type I and X collagens in comparison with the chondrogenic medium alone (Fig. 4B). On the opposite, heparin failed to affect the expression level of tested chondrogenic markers (Supporting Information Fig. 3).

### Interactions Between GY785 DR or GY785 DRS Polysaccharides and Growth Factors

**Figure 5:**
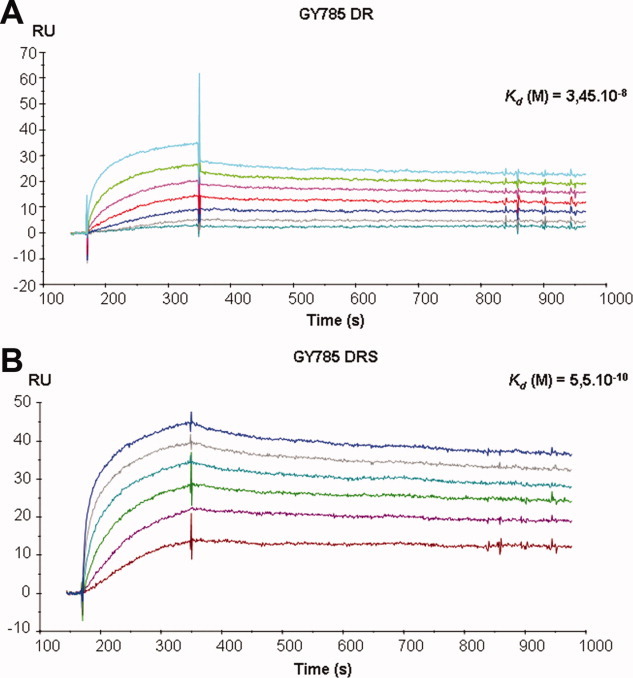
Determination of GY785 DR and GY785 DRS binding to transforming growth factor (TGF)-β1. The binding of GY785 DR **(A)** and GY785 DRS **(B)** to TGF-β immobilized on a research-grade CM5 chip was visualized as the change in surface plasmon resonance in the form of sensorgrams. The resulting sensorgrams were fitted using BiaEval 4.1 software. The regions used in determining equilibrium dissociation constants [*K*_d_ (M)] are shown. Abbreviation: RU, resonance unit.

To further address whether GY785 DRS may stimulate hATSC chondrogenesis, we then embarked on a set of surface plasmon resonance experiments. Chondrogenic medium contains two major constituents: TGF-β1 [22, 23] and insulin [24, 25], which are known to drive the chondrogenic differentiation of MSC. To investigate whether these factors can specifically interact with GY785 DR or GY785 DRS, quantitative measurements of their potential physical interaction were performed by Biacore analysis. Both GY785 DR and GY785 DRS polysaccharides were able to bind immobilized TGF-β1 (Fig. 5). By contrast, heparin was not able to bind these growth factors with a sufficient affinity to determine a *K*_d_ constant. The binding affinity of GY785 DR for TGF-β1 was approximately 100-fold lower than that of GY785 DRS polysaccharide, with a respective *K*_d_ of 3.45 × 10^−8^ and 5.5 × 10^−10^ M. No modification was observed in the kinetic parameters of the interaction between GY785 DR or GY785 DRS and immobilized insulin (data exhibiting a flat sensorgram are not shown). These results indicate that GY785 DRS can bind TGF-β1 with higher affinity than GY785 DR and neither of them interacts with insulin. Additional Biacore experiments were performed using BMP-2, VEGF, and IGF-1 as growth factors potentially secreted by MSC during their chondrogenic differentiation. Interestingly, we found that GY785 DRS interacts with BMP-2 and VEGF with respective *K*_d_ of 1.67 × 10^−9^ and 5.93 × 10^−10^ M, but not with IGF-1 (all *K*_d_ values are reported in Supporting Information Table 2).

### Effects of GY785 DRS on the TGF-β1 Signaling Pathway

**Figure 6:**
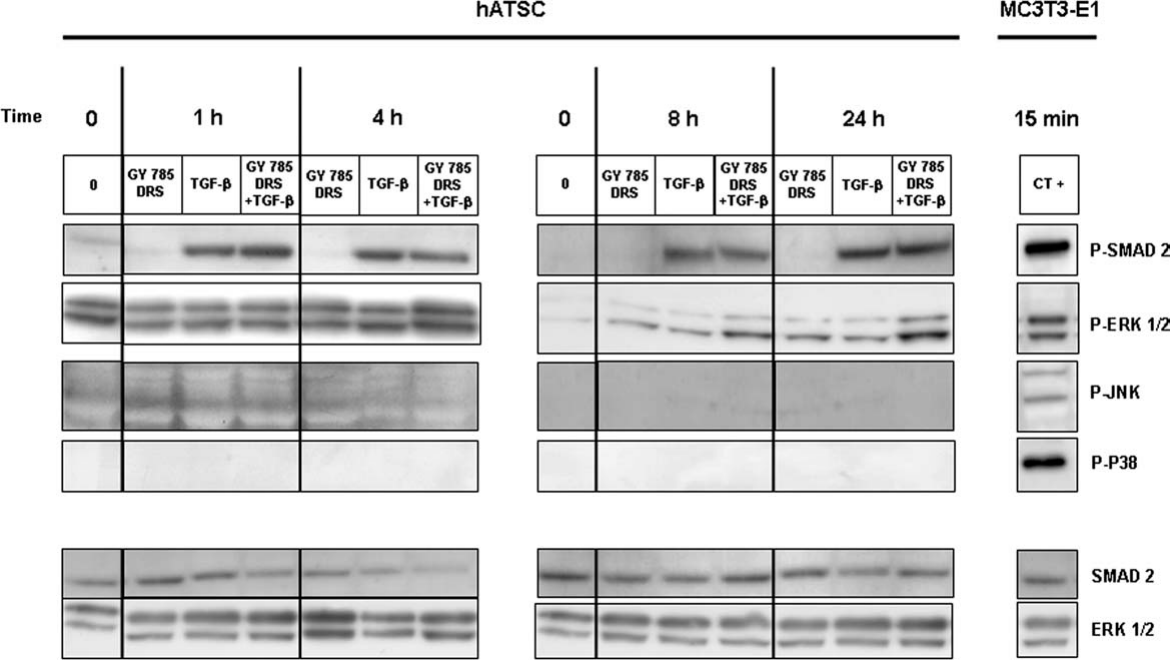
Effect of GY785 DRS on TGF-β1-dependent activation of SMAD 2 and mitogen activated protein kinase pathways. Human ATSCs were treated with 10 ng/ml of TGF-β1 in the presence or absence of 50 μg/ml of GY785 DRS for the indicated times. MC3T3-E1s were used as positive controls. Cells were rapidly frozen in liquid nitrogen before lysis on ice. The resulting samples were analyzed by Western blot using specific antibodies against P-SMAD 2, P-ERK1/2, P-JNK1/2, and P-p38, and/or antibodies against SMAD 2 and ERK1/2 as indicated. Data are representative of experiments with similar results. Abbreviations: CT, control; ERK, extracellular signal-regulated kinase; hATSC, human adipose tissue-derived mesenchymal stem cell; P-ERK, phosphorylated ERK; P-JNK, phosphorylated c-Jun N-terminal kinase; TGF, transforming growth factor.

To address whether the GY785 DRS/ TGF-β1 interaction demonstrated by surface plasmon resonance may lead to specific activation of cellular events in hATSC, we were interested in determining the potential upregulation of TGF-β-dependent activation of SMAD 2 in hATSC (Fig. 6) [26]. Immunoblots of cell lysates indicated that TGF-β1 alone or in association with GY785 DRS polysaccharide induced the phosphorylation of SMAD 2 as early as 1 hour and up to 24 hours. The presence of the sulfated polysaccharide alone was not sufficient to promote SMAD 2 activation and no additive or synergistic effect of GY785 DRS and TGF-β1 could be detected on the phosphorylation of SMAD 2. Since MAP kinase signaling pathways, including ERK, JNK, and p38, have been largely involved in TGF-β-dependent chondrogenesis [27, 28], we sought to decipher whether MAPK could be activated in response to treatment with TGF-β1 and GY785 DRS.

Of particular interest, whereas ERK1/2 phosphorylation was barely stimulated by TGF-β1 or GY785 DRS alone, the concomitant treatment of cells with TGF-β1 and GY785 DRS induced a marked upregulation of the phosphorylation of ERK1/2 as early as 4 hours. This stimulation was maintained up to 24 hours. Analysis of the phosphorylation of the other MAPK showed no detectable phosphorylation of either JNK or p38 in response to TGF-β1 and GY785 DRS treatment alone or combined. GY785 DR alone failed to affect any tested signaling pathway (data not shown).

To ensure the reliability of our detection method for the phosphorylation of SMAD 2, ERK1/2, JNK, and p38, MC3T3-E1 cells were treated with anisomycin, Pi, and TGF-β1 and used as a positive control. As expected, in these conditions, phosphorylation of p38 and JNK1/2 was observed after anisomycin treatment [21], phosphorylation of ERK1/2 was observed after Pi stimulation [21], and phosphorylation of SMAD 2 was observed in the presence of TGF-β1 [29] (Fig. 6 right panel).

## DISCUSSION

Injuries to articular cartilage are one of the most challenging issues of musculoskeletal medicine due to the poor intrinsic ability of this tissue for repair. Despite progress in surgery, the lack of efficient modalities of treatment for large chondral defects has prompted research into tissue engineering combining chondrogenic cells, scaffolding materials, and morphogenic factors. MSCs, initially isolated from bone marrow and extensively studied since, are an attractive source of cells for cartilage engineering. Over the past decade, MSCs from adipose tissue have been considered with a growing interest, due to their easy collection, their high capacity for in vitro expansion, and their chondrogenic potential [30]. However, MSC chondrogenic potential cannot be exploited without a considerable breakthrough in the use of adapted differentiation conditions, combining tridimensional culture and appropriate morphogens.

In this context, with a view to optimizing the chondrogenic differentiation of MSC for cartilage tissue regeneration, we were interested in identifying an easy-to-use, reliable, and safe chondrogenic agent.

A large body of evidence indicates that GAG-like polysaccharides can be involved in the regulation of various biological processes including cell adhesion, migration, proliferation, and differentiation [31], likely via the presence of sulfate groups on their carbohydrate structure. In light of the particular biological properties and chemical structure of oversulfated GAG-like polysaccharides of marine origin, we logically questioned whether GY785 DR and its oversulfated analog may affect MSC behavior.

It has been long described that sulfated GAGs, such as dermatan, chondroitin, and heparan sulfate, are able to stimulate the proliferation of fibroblasts [32], but the effects of marine sulfated polysaccharides on the proliferation of MSCs have not yet been characterized. Accordingly, in a first set of experiments, we were interested in demonstrating that the oversulfated GY785 DRS, and to a lesser extent its non-oversulfated isoform GY785 DR, have a slight but significant effect on viability and proliferation of hATSC. In addition to their effects on cell proliferation, we were then particularly captivated to observe that concomitant treatment of hATSC pellets with GY785 DRS (and not GY785 DR) and a growth factor-rich chondrogenic medium induced a dramatic increase in volume and GAG contents of chondrogenic pellets. Since the effects of GY785 DRS on DNA content were not significant, it is unlikely that the increase in pellet volume might be due to an increase in cell proliferation. Conversely, the increase in collagens and GAG contents and our immunostaining data allows us to speculate that the GY785 DRS was able to stimulate the production and accumulation of a chondrogenic matrix that could thus explain the increase in pellet volume. As GY785 DR and GY785 DRS are counterparts in structure and are only distinguishable by their degree of sulfation, our data clearly shed light on the role of the sulfate groups of polysaccharides in controlling their biological properties. Previous studies have reported that heparan sulfate and structurally related GAGs (dermatan sulfate and dextran sulfate) are involved in the regulation of chondrogenesis, specifically in nodule growth. Such studies have also found the nonsulfated forms to be quite less effective in stimulating chondrogenesis [33, 34].

To strengthen the scientific relevance of our histological findings, we then analyzed at the transcriptional level, whether GY785 DRS, together with our chondrogenic medium, was able to stimulate the expression levels of several chondrogenic genes. We focused our efforts on three extracellular articular cartilaginous matrix proteins type II collagen, aggrecan, and COMP, as well as on a transcription factor well known to drive the early step of MSC commitment toward the chondrogenic lineage, SOX9. Our real-time RT-PCR highlighted the upregulation of these four chondrogenic markers, thereby confirming our histological observations. Moreover, the presence of GY785 DRS in the chondrogenic medium was found to reduce the expression levels of unwanted transcripts coding for type I and X collagens and their corresponding proteins that are specific markers of osteogenesis and hypertrophy, respectively.

Sulfated GAGs were able to bind and regulate a number of proteins such as cytokines, chemokines, growth factors, morphogens, enzymes, and adhesion molecules. Some growth factors such as VEGF and FGF have been extensively described as being stored, stabilized, and protected from degradation in the matrix through interactions with GAG [35]. Under the action of a stimulus, these growth factors can then be released and exert their biological functions [36, 37]. These observations suggest that not only the binding affinity of a ligand to its receptor but also the stability of the ligand-receptor complex on the cell surface is one of the key factors that control the biological activity of the ligand in the targeted cells. To decipher whether TGF-β1 or insulin, the two major chondrogenic stimulatory factors of our culture medium, can physically interact with both GY785 DR and DRS, surface plasmon resonance experiments were performed. Interestingly, our Biacore data demonstrate the existence of a high affinity between GY785 DRS and TGF-β1, but not with insulin. On the contrary, the non-oversulfated isoform GY785 DR failed to exhibit such a high affinity for TGF-β1. Taken together, these findings shed further light on the possibility that the GY785 DRS may act synergistically with TGF-β1 through a physical interaction, as shown by the formation of affinity complexes in Biacore experiments.

Among its various biological effects, TGF-β1 is known to be a potent inducer of the osteochondrogenic differentiation of MSC by upregulating the SMAD and MAP kinase signaling pathways [27, 28]. We next investigated whether the in silico physical interaction seen by Biacore led to the activation of specific cellular responses in vitro. For this, we deciphered the SMAD and MAP kinase pathway activation in response to concomitant treatment of hATSC with GY785 DRS and TGF-β1. While the SMAD 2 pathway was not upregulated by this cotreatment, our experiments showed that GY785 DRS and TGF-β1 act concomitantly to induce a sustained activation of the ERK1/2 pathway. Collectively, our findings therefore strongly suggest that a physical interaction between TGF-β1 and GY785 DRS may be at the origin of the sustained ERK1/2 activation. Having demonstrated the existence of physical interactions between the GY785 DRS and particular growth factors, two putative mechanisms may explain the enhanced signaling observed within hATSC stimulated by the combined use of TGF-β1 and GY785 DRS. The first hypothesis is that GY785 DRS can protect growth factor to which it binds from proteolysis; thus, prolonging their lifespan and their biological activity.

The second hypothesis is that GY785 DRS can act as a coreceptor of growth factor and is participating in the stabilization of the interaction between the growth factor and its receptor, strengthening the intracellular signal arising from receptor activation.

## CONCLUSION

To conclude, we have demonstrated that GY785 DRS stimulates hATSC chondrogenic differentiation, probably through interaction with TGF-β1. This GAG-like polysaccharide of marine origin is easily producible and could therefore be considered a promising additive to drive efficient and reliable MSC chondrogenesis.

Whereas the underlying mechanisms of the interaction between GY785 DRS and TGF-β1 remain poorly understood, it could be of major relevance to further dissect these molecular mechanisms and their biological consequences, especially to help us monitor and exploit the potential of stem cells in cartilage repair.

## Supplementary material

Supplemental Figure 1 Structure of the nonasaccharidic repeating unit of GY785. GY785 DR is naturally sulphated at position C2 of the galacturonic acid residue in blue. GY785 DRS is a chemically oversulphated form of GY785 DR. Putative positions for additional sulphate groups (SO3Na) are indicated in red by arrows.

Supplemental Figure 2 Biochemical characterization of hATSC pellets Human ATSC were cultured in pellets in the presence of control (CT) or chondrogenic (CH) medium supplemented or not with 50 μg/mL of GY785 DR, GY785 DRS or low molecular weight heparin for 28 days. The pellet volumes were calculated by equating these entities to ellipsoids. * P<0.05 compared to the control condition. GAG production was evaluated at a gross level after alcian blue staining of the whole pellets. Bar: 500μm

Supplemental Figure 3 Analysis of the expression levels of chondrogenic markers. Human ATSC pellets were cultured in the presence of control (CT) or chondrogenic (CH) medium supplemented or not with 50μg/mL of GY785 DR, GY785 DRS or low molecular weight heparin for 28 days. Expression of the chondrogenic markers COL2A1 and ACAN was investigated by real-time RT-PCR. Results are expressed as relative expression level compared to the chondrogenic medium in the absence of polysaccharide. * p <0.05 compared to chondrogenic medium (CH).
