# Supplementary figures and images for: Pharmacological Modulation of Human Mesenchymal Stem Cell Chondrogenesis by a Chemically Oversulfated Polysaccharide of Marine Origin: Potential Application to Cartilage Regenerative Medicine
Source: Stem Cells. 2011 Nov 30;30(3):471–80. doi: 10.1002/stem.1686 (PMC3443367; doi:10.1002/stem.1686)

Figure 1  
(supplemental data)

TOP

J. Guicheux

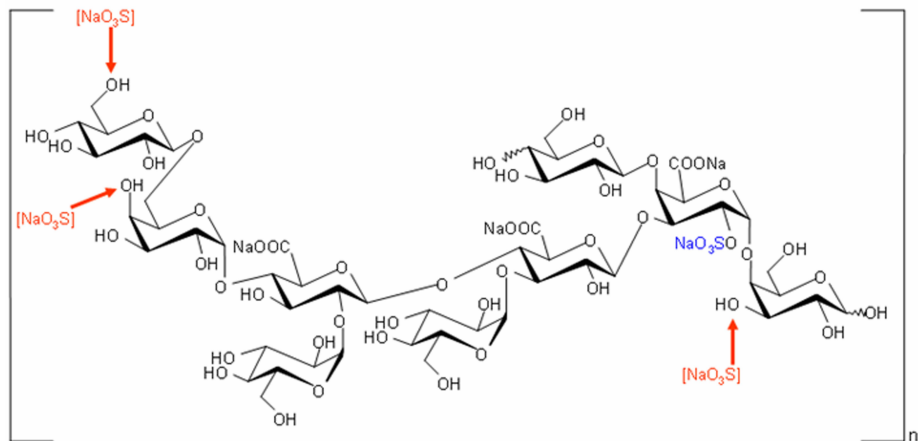

Supplement: Supplementary file 1 [file stem0030-0471-SD1.pdf]

## Supplemental data

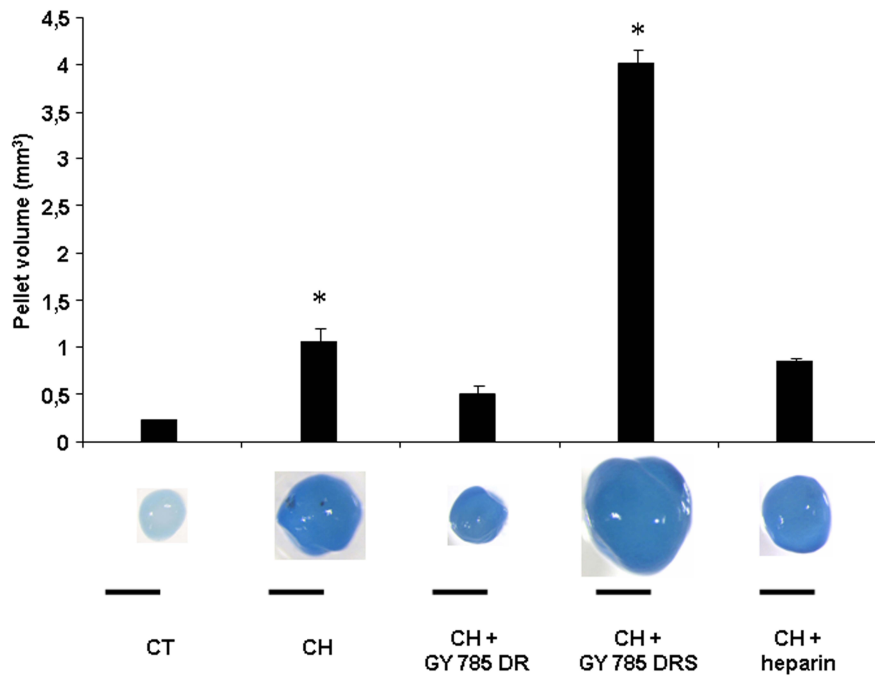

Supplement: Supplementary file 2 [file stem0030-0471-SD2.pdf]

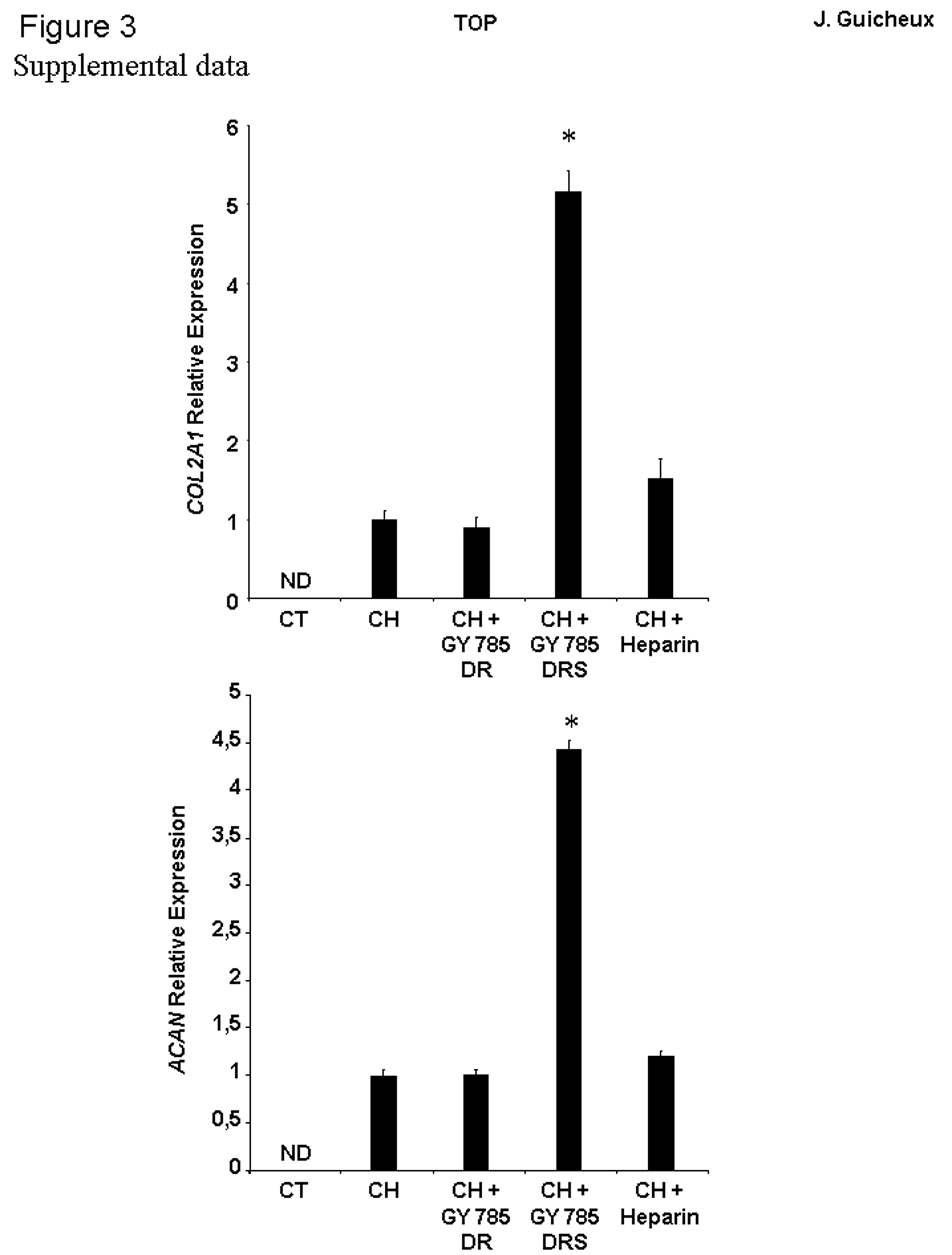

Supplement: Supplementary file 3 [file stem0030-0471-SD3.tif]
