# Supplementary material for: Pharmacological Modulation of Human Mesenchymal Stem Cell Chondrogenesis by a Chemically Oversulfated Polysaccharide of Marine Origin: Potential Application to Cartilage Regenerative Medicine
Source: Stem Cells. 2011 Nov 30;30(3):471–80. doi: 10.1002/stem.1686 (PMC3443367; doi:10.1002/stem.1686)
Supplement: Supplementary file 4 [file stem0030-0471-SD4.pdf]

Gene	Gene Bank Accession Number	Sequence	Base Pairs (bp)
β-ACTIN	NM_001101	Fwd 5'- CCAACCGCGAGAAGATGA -3'<br>Rev 5'- CCAGAGGCGTACAGGGATAG -3'	97
Type II collagen - α1 chain (COL2A1)	NM_001844	Fwd 5'- TGTCAGGGCCAGGATGTC -3'<br>Rev 5'- ATCATTATACCTCTGCCCATCC -3'	63
Aggrecan (ACAN)	NM_001135	Fwd 5'- CCTCCCCTTCACGTGTAAAA -3'<br>Rev 5'- GCTCCGCTTCTGTAGTCTGC -3'	64
Sex determining region Y-box 9 (SOX9)	NM_000346	Fwd 5'- GTACCCGCACTTGCACAAC -3'<br>Rev 5'- TCGCTCTCGTTCAGAAGTCTC -3'	72
Cartilage Oligomeric Matrix Protein (COMP)	NM_000095	Fwd 5'- GCACCGACGTCAACGAGT -3'<br>Rev 5'- TGGTGTTGATACAGCGGACT -3'	63
Type I collagen - α1 chain (COL1A1)	NM_000088	Fwd 5'- GGGATTCCCTGGACCTAAAG -3'<br>Rev 5'- GGAACACCTCGCTCTCCAG -3'	63
Type X collagen - α1 chain (COL10A1)	NM_000493	Fwd 5'- CACCTTCTGCACTGCTCATC -3'<br>Rev 5'- GGCAGCATATTCTCAGATGGA -3'	104

Table 1: Sequences of primer pairs, gene bank accession numbers used for real time RT-PCR analysis and size of PCR products.
