# Supplementary material for: Pharmacological Modulation of Human Mesenchymal Stem Cell Chondrogenesis by a Chemically Oversulfated Polysaccharide of Marine Origin: Potential Application to Cartilage Regenerative Medicine
Source: Stem Cells. 2011 Nov 30;30(3):471–80. doi: 10.1002/stem.1686 (PMC3443367; doi:10.1002/stem.1686)
Supplement: Supplementary file 5 [file stem0030-0471-SD5.pdf]

**Growth factors**	**GY785 DR K_d (M)**	**GY785 DRS K_d (M)**
TGF-β1	3.45×10^{-8}	5.5×10^{-10}
Insulin	-	-
BMP-2	1.09×10^{-7}	1.67×10^{-9}
VEGF	2.18×10^{-7}	5.93×10^{-10}
IGF-1	-	-

Table 2: K_d values determined by surface plasmon resonnance for the GY785 DR and its over-sulphated analogue, the GY785 DRS, in response to various growth factors exposure.
